# Effects of Starch Incorporation on the Physicochemical Properties and Release Kinetics of Alginate-Based 3D Hydrogel Patches for Topical Delivery

**DOI:** 10.3390/pharmaceutics12080719

**Published:** 2020-07-31

**Authors:** Sara Bom, Catarina Santos, Rita Barros, Ana M. Martins, Patrizia Paradiso, Ricardo Cláudio, Pedro Contreiras Pinto, Helena M. Ribeiro, Joana Marto

**Affiliations:** 1 Research Institute for Medicines (iMed.ULisboa), Universidade de Lisboa, 1649-003 Lisbon, Portugal; sarabom@campus.ul.pt (S.B.); amartins@farm-id.pt (A.M.M.); geral@phdtrials.com (P.C.P.); hribeiro@campus.ul.pt (H.M.R.); 2 PhD Trials, Avenida Maria Helena Vieira da Silva, n° 24 A, 1750-182 Lisbon, Portugal; 3 EST Setúbal, CDP2T, Instituto Politécnico de Setúbal, Campus do IPS-Estefanilha, 2910-761 Setúbal, Portugal; catarina.santos@estsetubal.ips.pt (C.S.); ricardo.claudio@estsetubal.ips.pt (R.C.); 4 CQE, Instituto Superior Técnico, Universidade de Lisboa, Av. Rovisco Pais 1, 1049-001 Lisboa, Portugal; patrizia.paradiso@tecnico.ulisboa.pt; 5 Faculty of Pharmacy, Universidade de Lisboa, 1649-003 Lisboa, Portugal; ritabarros97@hotmail.com; 6 IDMEC, Instituto Superior Técnico, Universidade de Lisboa, Av. Rovisco Pais 1, 1049-001 Lisboa, Portugal

**Keywords:** 3D printing, alginate, starch, quality by design, skin delivery

## Abstract

The development of printable hydrogel inks for extrusion-based 3D printing is opening new possibilities to the production of new and/or improved pharmaceutical forms, specifically for topical application. Alginate and starch are natural polysaccharides that have been extensively exploited due to their biocompatibility, biodegradability, viscosity properties, low toxicity, and relatively low cost. This research work aimed to study the physicochemical and release kinetic effects of starch incorporation in alginate-based 3D hydrogel patches for topical delivery using a quality by design approach. The incorporation of a pregelatinized starch is also proposed as a way to improve the properties of the drug delivery system while maintaining the desired quality characteristics. Critical material attributes and process parameters were identified, and the sensitivity and adequacy of each parameter were statistically analyzed. The impact of alginate, starch, and CaCl_2_·2H_2_O amounts on relevant quality attributes was estimated crosswise. The amount of starch revealed a synergetic impact on porosity (*p* = 0.0021). An evident increase in the size and quantity of open pores were detected in the *as printed* patches as well as after crosslinking (15.6 ± 5.2 µm). In vitro drug release studies from the optimized alginate-starch 3D hydrogel patch, using the probe Rhodamine B, showed an initial high burst release, followed by a controlled release mechanism. The results obtained also showed that the viscoelastic properties, printing accuracy, gelation time, microstructure, and release rates can be modulated by varying the amount of starch added to the system. Furthermore, these results can be considered an excellent baseline for future drug release modulation strategies.

## 1. Introduction

Three-dimensional (3D) printing is an additive manufacturing process that unraveled new and quite promising possibilities for the pharmaceutical and medical communities; 3D printing applications are revolutionizing health care in different categories including tissue and organ fabrication, prosthetics, implants and anatomical models, and research in drug delivery dosage forms [1]. The versatility of this process is also opening novel prospects to produce new and/or improved pharmaceutical dosage forms, especially for topical application. This unique technology uses computer-aided drafting technology and programming to generate 3D solid or semi-solid objects of virtually any shape from a 3D model file. The 3D models can be generated by computer aided design (CAD) software or obtained from 3D scanners [1,2,3,4]. There are different printing strategies such as powder-based printing, extrusion-based printing (fused deposition modelling (FDM) and pressure-assisted microsyringes (PAM)), stereolithographic printing, selective laser sintering printing, inkjet printing, and digital light processing [5]. Among these, extrusion-based PAM printing has recently gained popularity in pharmaceutical and cosmetic applications due to the ability to print semi-solid formulations (gels or pastes) at room temperature; to print using a wide range of polymers with or without drug; and to modulate drug release by tuning the geometry, type, and amount of polymer [4,5]. Despite being considered a very controlled, advantageous, and sustainable process, it is necessary to understand which critical parameters are associated with the 3D printing process to guarantee the quality of the final product [6]. During the process, several parameters such as needle size, deposition speed, and ink viscosity must be controlled. Additionally and as recently mentioned by Azad et al. [5], it is fundamental to understand which polymers are suitable for extrusion-based 3D printing, to determine the interactions between the polymer and the therapeutic substances, and to understand how their properties impact the printing process.

In this context, the formulation of natural and/or synthetic polymeric hydrogels for extrusion-based 3D printing has been studied. The choice of polymeric materials is essential to ensure the development of suitable 3D constructs. Alginate and starch are natural polysaccharides that have been extensively exploited due to their biocompatibility, biodegradability, viscosity properties, low toxicity, and relatively low cost [7,8]. The chemical structure of alginate (linear co-polymer) consists of two blocks structures, β-d-mannuronic acid (block M) and α-l-guluronic acid (G block), which are linked by α-(1, 4) glycosidic bonds. The presence of carboxyl groups in the chemical structure allows the alginate to crosslink with di- or trivalent metal cations (Ca^2+^, Fe^2+^, Mn^2+^, and Al^3+^), with Ca^2+^ being commonly used to produce high-quality gels or films [8]. Cations pull alginate chains together via ionic interactions, after which interchain hydrogen bonding occurs [9]. Starch is composed of two polymers of α-d-glucopyranose: the linear amylose and the highly branched amylopectin. The presence of free hydroxyl groups in its structure confers a synergism in composite hydrogels (i.e., hybrid systems composed of two or more components that are chemically or physically linked) that can improve water absorption capacity, encapsulation efficiency, and controlled release of therapeutic substances [8]. Furthermore, starch is chemically versatile (depending on the source) and can be modified to obtain high quality mechanical and rheological properties [10]. In food manufacturing studies, starch has been shown to be a suitable polymer for extrusion-based 3D printing, providing good printability [11,12,13,14]. Other studies also have reported that the addition of starch in alginate-based 3D systems improved the performance and quality of the final products. Singh et al. [15] developed starch-alginate beads for controlled release of pesticides and concluded that release rate increased with increasing starch content. Córdoba et al. [16] developed an alginate-starch composite hydrogel loaded with yerba mate antioxidants and observed that the addition of starch significantly improved the encapsulation efficiency of the extract from 55 to 65%, modulated and controlled the antioxidants release rate, and decreased alginate erosion. Hosseini et al. [17] studied the effect of nisin encapsulation in alginate and alginate-resistant starch microparticles and obtained data suggesting that the introduction of resistant starch in alginate microparticles resulted in higher encapsulation efficiency and loading capacity values. Lozano-Vasques et al. [18] studied the effect of the weight ratio of alginate-modified tapioca starch on the physicochemical properties and release kinetics of chlorogenic acid-containing beads. The authors observed that calcium alginate beads with increasing tapioca starch content achieved increasingly higher chlorogenic acid encapsulation efficiency. In addition, the results showed that the microstructure, textural characteristics, viscoelastic properties, chlorogenic acid release rates, and antioxidant stability could be modulated by the amount of starch added to the system. Fernandes et al. [8] developed alginate-starch composite hydrogels crosslinked with different ions (Ca^2+^, Zn^2+^, and Mn^2+^) to evaluate the effect of different concentrations and crosslinking agents on hydrophilic, kinetic, and spectroscopic properties. The results showed that the increase of starch concentration led to an increase in the swelling degree of the hydrogels and that the different crosslinks studied altered the water absorption capacity, the mechanism, and velocity of water uptake from the hydrogels.

The study reported herein aimed to develop an alginate-pregelatinized starch drug delivery system (DDS) for topical application resorting to an extrusion-based PAM printing process. The formulation and process parameters were optimized using a Quality by Design (QbD) approach. The different factors that can affect gelation time, porosity, construct integrity, and spreadability during the printing process were evaluated, including the percentage of alginate, CaCl_2_ dihydrate, and starch. Finally, the in vitro Rhodamine B release profile and kinetics were determined.

## 2. Materials and Methods

### 2.1. Materials

Alginic acid sodium salt powder (medium-viscosity: ≥2000 cP), calcium chloride dihydrate (CaCl_2_·2H_2_O), and Rodhamine B (purity degree ≥ 95%) were purchased from Sigma-Aldrich^®^ (St. Louis, MO, USA). Low-viscosity pregelatinized modified starch (Instant Pure-Cote^®^ B793-NF) was obtained from Paroxite (London, UK). Colloidal silver was purchased from Argenol (Zaragoza, Spain). Purified water was obtained by reverse osmosis and electrodeionization (Millipore^®^, Elix 3), followed by filtration (filter pore 0.22 µm) and sterilization.

### 2.2. Methods

#### 2.2.1. Identification of Quality Target Product Profile (QTPP) and Critical Quality Attributes (CQAs)

The quality target product profile (QTPP) describes the desired quality profile, and it was used as the base to identify the CQAs, the critical material attributes (CMAs), and the critical process parameters (CPPs). CMAs and CPPs are defined as the inputs which, by appropriate selection and interaction, will deliver the output. Therefore, the first step consisted of defining the quality attributes of the product: 3D hydrogel patch. The desired QTPP depends upon scientific, regulatory, and practical considerations and limitations. The most important features of 3D hydrogel patches have been defined as gelation time, porosity, printing accuracy, elasticity, and adhesiveness. The list of the key QTPP is presented in Table 1.

#### 2.2.2. Risk Analysis of CQAs

An Ishikawa diagram was used to identify and list the potential risks that may influence the 3D hydrogel patch printing process using a 3D Focus printer (ByFlow Eindhoven, The Netherlands) by a semi-solid PAM printing method. This approach enabled the identification of the CQAs that have the greatest chance of leading to product failure, while also allows prioritizing the possible risk factors associated with the CMAs and CPPs. In addition, a Risk Estimation Matrix (REM) was designed to rank and prioritize the potential low-, medium- and high-risk material attributes and to process parameters previously identified. Severity (S), Occurrence (O), and Detection (D) of potential failures that may occur during the product lifecycle were determined using a numerical scale from 1 to 5, with 1 being the lowest severity, probability, and undetectability and 5 the highest. The risk estimation matrix used for values attribution is shown in Figure S1 (Supplementary Materials). Risk Priority Number (RPN), which helps prioritize risks or actions for problem resolution, was calculated using the following equation:

(1) RPN=S×O×D

#### 2.2.3. Alginate-Starch Hydrogel Inks

Alginate (Alg) hydrogels were prepared by dissolving alginic acid sodium salt in purified water in concentrations ranging from 1.5% to 4.5% (*w*/*v*). Alginate-starch (Alg-St) hydrogels were prepared with ratios of 1.5:4, 3:2 and 4.5:4 (*w*/*w*). The final ink viscosity was considered as the most influencing parameter for the Alg-St ratio’s selection. All formulations were magnetic-stirred overnight (12 h) to obtain homogeneous hydrogels. The cross-linking solutions were calcium chloride dihydrate (CaCl_2_·2H_2_O) in purified water, with concentrations ranging from 0.7% to 3.0% (*w*/*v*). Colloidal silver was incorporated at a concentration of 0.1% for increased contrast in confocal images. For the release studies, Rhodamine B was incorporated at the recommended concentration of 0.05%, and the formulations were kept in the dark at 2 °C before printing.

#### 2.2.4. Physicochemical Characterization of Alginate-Starch Hydrogel Inks

Viscosity measurements were performed using a controlled stress Kinexus Rheometer (Malvern Instruments, Malvern, UK) employing a cone-and-plate geometry (truncated angle 4° and radius 40 nm). The measurements were performed between 1 and 1000 Pa on a logarithmic increment, ranging from 0.1 to 100 s^−1^. Oscillation frequency sweep tests were performed at frequencies ranging between 0.01 and 1 Hz, using the same cone-and-plate geometry. In order to determine the gelation time, a single frequency strain-controlled time event sequence was designed. The measurements were carried out with a plate–plate geometry and a gap of 0.5 mm (patch height simulation). Hydrogel inks were mixed with the crosslink solution at a ratio of 1:10 and vortexed for 30 s before sample loading to simulate a non-saturation gelation process. The onset of gelation time was recorded as the time when a minimum of 7 successive data points showed the sol/gel transition point. All measurements were performed at 25 °C.

#### 2.2.5. 3D Printing Process

The 3D Focus printer (ByFlow, Eindhoven, The Netherlands) was used to evaluate the printability of the generated inks. All hydrogels were printed using a 0.8-mm needle. The structure corresponds to a pad with 20 mm × 20 mm × 0.5 mm. Printed patches were generated with Ultimaker Cura slicing software, version 4.4 (Utrecht, The Netherlands). The geometry was sliced in two layers (0.25 mm each, including the first one) and printed at first with two contours and the middle filled at 100% with directions of ±45°. The feed rate was defined as constant for all the contours and experiments (10 mm/s). The printing parameters were adjusted considering the rheological properties of the different Alg and Alg-St aqueous solutions. The polymeric aqueous dispersions were printed and the CaCl_2_·2H_2_O solution was added immediately after (ionic cross-linking method). Glass was selected as the support material to improve adhesion during patch printing.

#### 2.2.6. Formula Optimization and Establishment of Design Space

The formula of the Alg-St 3D patches was optimized using a Full Factorial Design (FFD), composed by 3 levels (−1, 0, and +1). The data were statistically analyzed using the MODDE^®^ software (Umetrics, Umeå, Sweden), and differences were considered significant for *p* < 0.05. According to preliminary studies and risk assessment analysis, the percentages of alginate (X_1_), CaCl_2_·2H_2_O (X_2_) and starch (X_3_) were defined as the factors to be analyzed, while gelation time (Y_1_), porosity (Y_2_), construct integrity (Y_3_), and spreadability ratio (Y_4_) were defined as the responses. This design required 11 experimental runs, including three replicated center points for the estimation of the prediction variance over the entire design space (see the Supplementary Materials, Table S1). The following mathematical model was fitted to the data:

(2) $$Y_{n}=\beta_{0}+\beta_{1}X_{1}+\beta_{2}X_{2}+\beta_{3}X_{3}+\beta_{12}X_{1}X_{2}+\beta_{13}X_{1}X_{3}+\beta_{23}X_{2}X_{3}$$

where Y denotes the response associated with each factor level combination; β_0_ depicts the arithmetic average; β_1_, β_2_, and β_3_ represent the first order coefficients of the respective independent variables; and β_12_, β_13_, and β_23_ typify the interaction coefficients. The positive and negative signs of the coefficient values indicate a synergetic or antagonistic effect of each term, respectively, while the magnitude represents the impact extent. An analysis of variance (ANOVA) was also performed to statistically analyze the fitted models.

#### 2.2.7. Hydrogel 3D Patches Characterization

The optimized Alg-St 3D patch was characterized. The alginate-3D patch without starch (Alg) was used as the control; the concentration of crosslink solution was the same (1.7%) for both inks.

##### Gelation Time, Porosity, and Construct Integrity

Gelation time was recorded during the design assay, and the macroscopic properties of the Alg and Alg-St hydrogels were visually analyzed. The time at which the gel did not flow/spread was recorded as the gelation time (minutes).

The porosity was analyzed using an optical microscope (Leica DM2500, Wetzlar, Germany). The images (1980 × 960) were acquired at 400× magnification, and the diameter of the pores (*n* = 50) was measured using ImageJ^®^ software.

The construct integrity during printing was evaluated by measuring the spreading behavior–area (mm^2^) before and after gelation. The area of the printed patches was measured using ImageJ^®^ software.

##### Spreadability

A S-shaped filament with 0.8 mm × 22 mm (z = 1.5 mm; distance between lines = 6.8 mm) was used to evaluate the spreading behavior during the printing and gelation process. The width of the printed filament was measured using ImageJ software^®^ 1.52v (NIH, Bethesda, MD, USA) and divided by the internal needle diameter to obtain the spreading ratio. The printing accuracy (PA) was evaluated by measuring the spreading behavior in percentage.

(3) $$\mathrm{Spreading}\;\mathrm{ratio}=\frac{\mathrm{Printed}\;\mathrm{needle}\;\mathrm{diameter}}{\mathrm{Needle}\;\mathrm{diameter}}$$

##### Physicochemical Characterization

The morphology of the 3D patches was examined using a scanning electron microscope (FEG-SEM JEOL 7001F, JEOL, Tokyo, Japan). To increase the conductivity, the 3D patches were coated with a thin layer of gold/palladium and examined using an acceleration voltage of 25 kV.

The surface profiles and topography measurements were obtained using an Optical profilometer Profilm 3D (Filmetrics, San Diego, CA, USA). The images were acquired with a 50× magnification. This assay was performed 7 days after printing to ascertain the stability of the patches. The patches were stored in dry conditions at 2 °C and protected from the light. Confocal images (Vivascope 1500, Caliber I.D.) were recorded and used to assess the 2D morphology and to construct the 3D patches structure resorting to 3D Slicer software. The images were imported to the software in Tiff format, and the correct image spacing and origin values were set in the volume information section. In the editor section, the pore area was selected by adding a structure and by applying the “Threshold effect”. Therefore, the 3D structure was created resorting to the “Model Maker” tool in “Surface models”. In this assay, colloidal silver was incorporated into the ink to increase contrast. The pore size diameter of the 3D patches was analyzed using an optical microscope (Leica DM2500, Wetzlar, Germany). Images (1980 × 960) were recorded at 400× magnification. The diameter of the pores (*n* = 150) was measured using ImageJ^®^ 1.52v (NIH, Bethesda, MD, USA). The pore size distribution was determined using a Microsoft Excel-based workbook and Add-in software.

To identify the functional groups, attenuated total reflectance (ATR-FTIR) spectra were acquired using a Nicolet FTIR spectrometer (Thermo Electron, Thermo Fisher Scientific, Waltham, MA, USA); 3D patches and respective raw materials were placed on the ATR diamond crystal, and spectra were obtained in the range of 4000 to 500 cm^−1^, resulting in an average of 128 scans collected with a resolution of 8 cm^−1^.

#### 2.2.8. In vitro Release Studies

For the release experiments, 3D patches with 10 mm × 10 mm × 0.5 mm (*n* = 6, for each assay) loaded with Rhodamine B (model drug) were suspended in 2 mL of Phosphate Buffered Saline (PBS) solution at room temperature and static conditions. Samples (200 μL) were collected from the receptor phase at 0, 2.5, 5, 10, 15, 30, 60, 120, 180, 240, 300, and 360 min, and the volumes were replaced with fresh medium at the same temperature. The absorbances of the samples were measured in a Fluostar Omega microplate reader (BMG Labetch, Ortenberg, Germany) at 580 nm (maximum emission wavelength of Rhodamine B). The percentage of Rhodamine B released into the medium was calculated using the following equation:

(4) $$\mathrm{Cumulative}\;\mathrm{release}\;\mathrm{percentage}\;=\overset{t}{\underset{t=0}{\sum }}\frac{M_{t}}{M_{0}}\times 100$$

where *M_t_* is the cumulative amount of Rhodamine B released at each sampling time point, *t* is time, and *M*_0_ is the initial weight of the Rhodamine B in the Alg-St patches.

The release studies were performed five times, and mean values of cumulative release (%) were plotted against time. The data obtained from in vitro release studies were computed using DDsolver [24], an Excel-plugin module, and different kinetic models were fitted to the resultant data:

(1) Zero-order kinetics
  $$F=K_{0}\times t$$
  where *K*_0_ is the zero-order release constant
(2) First-order kinetics
  $$F=100\times \left(1-e^{-K_{1}\times t}\right)$$
  where *K*_1_ is the first-order release constant.
(3) Higuchi model
  $$F=K_{H}\times t^{1/2}$$
  where *K_H_* is the Higuchi release constant.
(4) Korsmeyer–Peppas model
  $$F=K_{KP}\times t^{n}$$
  where *K_KP_* is the release constant incorporating structural and geometric characteristics of the drug-dosage form and *n* is the diffusional exponent indicating the drug-release mechanism.
(5) Weibull model
  $$F=1-\exp\left[\frac{-{\left(t-T_{i}\right)}^{\beta }}{\alpha }\right]$$
  where *α* defines the time scale of the process; $T_{i}$ represents the lag time before the onset of the dissolution or release process; and *β* is the shape parameter which characterizes the curve as either exponential (*β* = 1), sigmoid, S-shaped, with upward curvature followed by a turning point (*β* > 1), or parabolic, with a higher initial slope and after that consistent with the exponential (*β* < 1).

In all models, *F* is the fraction (%) of released drug at time *t*. The adjusted coefficient of determination (R^2^_adjusted_) was estimated for each model and used as an estimate of the goodness-of-fit, i.e., the model ability to describe a given dataset. The R^2^_adjusted_ values and the Akaike minimum information theoretical criterion (AIC) were used as a measure of fit to compare the different models. When comparing several competing models, the best fitting model is the one with maximum $R_{\mathrm{adjusted}}^{2}$ value and minimum AIC value.

## 3. Results and Discussion

### 3.1. Risk Analysis of CQAs

#### 3.1.1. Process Mapping

**Figure 1:**
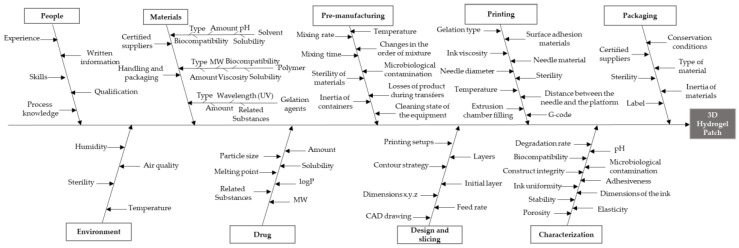
Ishikawa diagram illustrating factors that may have impact on the development of a 3D hydrogel patch.

An Ishikawa diagram, also known as fishbone diagram, is one of the seven basic tools of quality management. This tool was used to establish which variables could have an impact on the desired quality of the product. First, a brainstorm session was performed to identify and list potential risk parameters. Subsequently, the list was reviewed to extract relevant causes in the context of the main problem, the 3D hydrogel patch, which is represented in the “fish head”. The potential causes of the problem, derived from the brainstorming session and research, were then organized in the “fish bones” of the Ishikawa diagram (Figure 1). A total of nine potential causes were listed: people, environment, materials, drug, pre-manufacturing, design and slicing, printing, characterization, and packaging. Each factor was subdivided to show the relationship of all potential causes to the presenting problem.

#### 3.1.2. Risk Estimation Matrix (REM)

In this study, a QbD approach was employed to extract the maximum knowledge from the data collected and to establish the influence of several factors on the formulation and physicochemical properties of Alg-St 3D patches for topical application.

When using QbD to optimize formulation and process development, the first step is to predefine the desired final QTPP. This study focused on the critical formulation- and process-dependent quality attributes that mostly influence the quality of 3D hydrogel patches for topical application: gelation time, porosity, printing accuracy, elasticity, and adhesiveness. Gelation time was set at 10–30 min since previous studies have shown that a rapid gelation time leads to nonuniform crosslinking, which in turn can have a negative impact on the rheological and mechanical properties of the final product [19]. Considering the typical pore size of alginate (3–17 nm/5–21 µm, depending on the analysis technique [21]) and starch (0.5–8 µm, depending on the starch source [20]) when used as single polymeric components, porosity was set to 1–20 µm to guarantee a controlled drug delivery. Printing accuracy was established as high since this feature is fundamental to guarantee the bulk structure stability [22]. Elasticity and adhesiveness of the 3D hydrogel patch were defined according to the application area.

Risk analysis studies allow the reduction of the number of experiments needed to identify the most critical factors that affect the response under evaluation. Potential CQAs were derived from the QTPP and used to construct a REM considering the CMAs and CPPs that were selected from the Ishikawa diagram. The REM was designed to ascertain which raw material attributes and process parameters needed further study and control (Table 2). The exact values attributed for the different parameters are presented in Table S2 (Supplementary Materials).

The risk attribution for raw materials attributes and process parameters was based on literature and previous knowledge. Considering the raw materials attributes, the data obtained highlight the need to control the amount of water, alginate, and starch as well as the concentration of CaCl_2_ dihydrate. Porosity and printing accuracy were considered the CQAs with the greatest probability of generating product failure when considering the amount of solvent, polymer, and crosslink. To reduce the impact of the raw materials on the CQAs, medium viscosity alginate and pregelatinized modified starch were selected. As shown in previous studies, the use of a pregelatinized modified starch results in clear and stable dispersions with low polymer concentrations and eliminates the need for heating since this polymer is cold-water soluble [25].

The influence of the process variables that determine the characteristics of ionic cross-linked Alg-St 3D patches was also investigated. These include mixing time and rate, printing setups, dimensions (x, y, z), number of layers and contour strategy, feed rate, needle material and diameter, distance between the needle and the platform, ink viscosity, surface adhesion materials, and extrusion chamber filling. Mixing time and rate have a medium impact on the gelation time, porosity, and printing accuracy, which is related to the air that is introduced in the mixing and to the dispersion of the polymer in the aqueous solution. According to Cohen et al. [26], increasing the mixing process reduced material inconsistency and improved geometric fidelity and stability of 3D alginate hydrogels. Piskounova et al. [27] showed that insufficient mixing led to softer and not fully crosslinked hydrogels, while excessive mixing led to weaker hydrogels most likely due to defects in the 3D network. Dimension, number of layers, and contour strategy were considered to have a medium risk for gelation time, porosity, and printing accuracy since these parameters have a direct influence on the spreadability ratio during printing which will have an impact on construct integrity after printing. Considering the 3D slicing-printing process and, according to the literature, air pressure related to the extrusion chamber filling, feed rate, and printing distance (i.e., distance between the needle and the platform) are the most important factors which can influence the printing quality. These parameters can be adjusted by reducing the extrusion rate or by accelerating the moving speed [22]. These observations were considered when assigning low risk to such parameters. Surface adhesion materials were assigned medium risk for printing accuracy since bed adhesion is interlinked with the wettability properties of the hydrogel ink and could drastically influence the quality of the final product. Regarding needle diameter, a smaller needle size results in higher printing resolution, as pertained in QTPP [28]. However, the needle diameter can influence the ink viscosity during printing. Moreover, ink viscosity was assigned as having medium impact on gelation time and printing accuracy and as high risk on porosity, elasticity, and adhesiveness. Ink viscosity can be adjusted through an adequate polymer concentration selection, but the impact on the quality of the final product needs further investigation.

Therefore, the medium- and high-risk parameters were selected for subsequent screening studies to accurately establish the interaction between the raw materials and the 3D hydrogel patch CQAs.

#### 3.1.3. Adjustment of CPPs

Considering the CPPs, preliminary studies were designed to adjust the experimental conditions in function of the raw materials interactions. Thus, the mixing time was adjusted to 12 h to guarantee the development of homogenous formulations, and mixing rate was adjusted considering the viscoelastic properties of the formulations. The final structure corresponds to a pad with 20 mm × 20 mm × 0.5 mm. The geometry was sliced in two layers of 0.25 mm each and printed at first with two contours and the middle filled at 100% with directions of ±45° to guarantee the construct integrity and bulk structure. Furthermore and according to preliminary studies, a 0.8-mm needle was selected to minimize the impact on the ink viscosity during printing. A delay between layers printing was also considered, since samples printed with a higher delay between layers have higher dimensional accuracy and are significantly closer to CAD software-based designs [29]. Different support materials (agar-agar, plastic, and glass) were studied to improve adhesion during patch printing. Among these, glass resulted in more accurate printing (see the Supplementary Materials, Figure S2).

### 3.2. Effect of Starch on Physicochemical Properties of the Alginate-Starch-Based Inks

**Figure 2:**
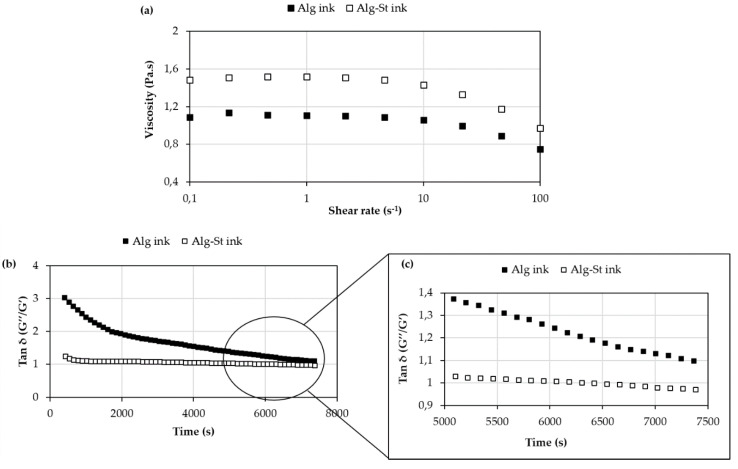
Evaluation of alginate-starch-based inks rheological properties, before (**a**) and during gelation (**b**,**c**).

The properties of the ink before, during, and after gelation are essential for its printability, comprising such features as achievable structural resolution and shape fidelity. Thus, the rheological behavior, before and during the printing process, is considered a fundamental parameter of analysis to understand the relation between the polymer(s) and the crosslink solution, and its influence on gelation time and printing accuracy. As shown in Figure 2a, both inks exhibited a similar behavior to the torque response (i.e., similar flow curve) before gelation, while the introduction of starch caused a slight increase in apparent viscosity.

Considering the rheological results obtained before gelation, it would be expected that an increase in viscosity would lead to a slower rearrangement of the conformations of the polymer chain (longer gelation time). However, a detailed assessment of the interaction between the elastic and viscous modules may be more accurate to draw such conclusions. Tan δ (G’’/G’), the loss or damping factor, is often used to describe the viscoelastic behavior in samples when there is a phase transition [30,31]. In rheology, tan (δ) quantifies the balance between energy loss and storage. The fact that G’ increases at higher time means that structural changes took place. In Figure 2b,c, Tan δ (G’’/G’) is presented as a function of time sequence. As previously mentioned in Section 2.2.4. the onset of gelation time was recorded as the time when a minimum of 7 successive data points showed the sol/gel transition point. It can be observed that a decrease of Tan (δ) values revealed that both materials act more elastic than viscous, having more potential to store the load rather than dissipating it. Nevertheless, during gelation, the elastic (G’) and viscous modulus (G’’) of the Alg-St ink were similar and lower than the corresponding values obtained for the Alg ink. For Alg ink, G’ and G’’ were significantly different, with the viscous module being significantly higher than the elastic module (Tan (δ) > 1). The results show that the addition of starch has a direct influence on the elastic properties of the printed patch, which is reflected in a shorter gelation time (6780 s) compared to the Alg ink (> 7200 s), Figure 2c. In fact, only in Alg-St ink it was observed a phase transition at 7000 s, (Tan (δ) <1), (Figure 2c). This means that the character of the sample has changed during the measurement from the liquid to the solid or gel state. The data corroborate the results reported by Viyoch et al. [32], which showed that the addition of starch can improve the elasticity of the printed patch. However, a faster gelation time can affect the structural integrity of the construct. Furthermore, the non-saturation of the polymeric system led to a nonuniform distribution of calcium ions through the system, which induced the formation of large clusters, maximizing the formation of heterogeneous gelled networks. This type of process also increased the gelation time. Thus, in subsequent studies, gelation was induced by saturation of the system.

The oscillatory data was also used to infer the adhesiveness strength of the hydrogel inks. Considering G’ > G’’ values in Alg-St ink, it conferred higher adhesive properties for topical application comparing to the Alg ink. The higher swelling behavior attributed to starch-based hydrogels can possibly contribute to an improved bioadhesive behavior [8].

### 3.3. Formula Optimization and Establishment of Design Space

**Figure 3:**
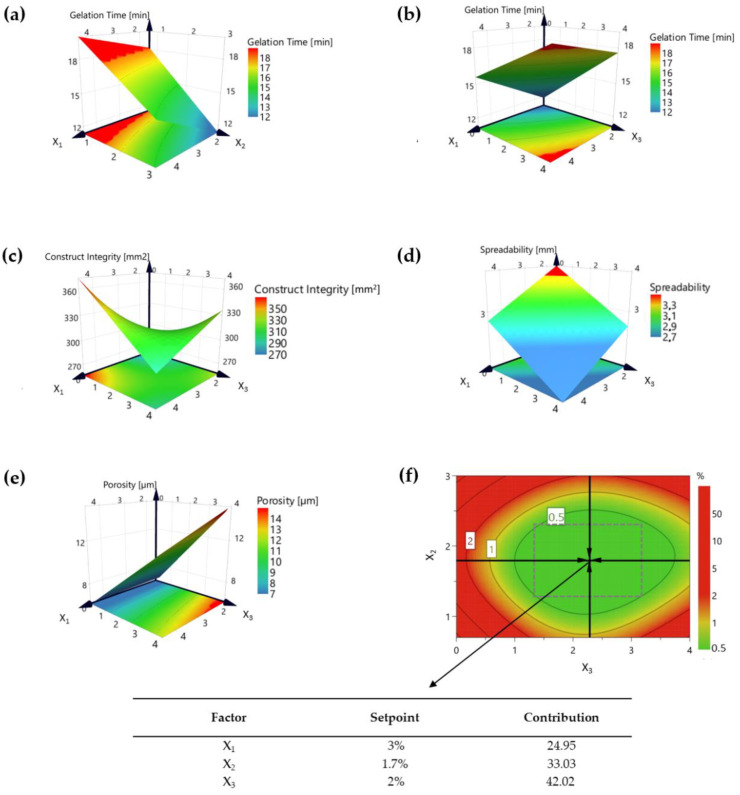
Response contour plots of the fitted model for gelation time (**a**,**b**), construct integrity (**c**), spreadability (**d**), porosity (**e**), and design space (**f**).

The choice of variables under analysis is a task of paramount importance because it limits both results and interpretation. REM analysis and preliminary results clearly evidenced that 3D Alg-St hydrogel patches are highly influenced by the polymers and CaCl_2_·2H_2_O concentrations. After selecting the most important factors influencing the physicochemical properties, an optimization study was performed to identify and establish the interaction between the raw materials and the 3D hydrogel patch CQAs. Specifically, the impact of alginate (X_1_), CaCl_2_·2H_2_O (X_2_), and starch (X_3_), and their interactions on predefined CQAs were studied. At different factor levels combinations, a total of 11 runs (including 3 center replicates) were performed. To evaluate the effect of X_1_, X_2_, and X_3_ variables on 3D alginate-starch hydrogels patches, Design of Experiments (DoE) patches were characterized for the main quality attributes. The data obtained by the experimental designs were analyzed with the MODDE^®^ software, and first-order polynomial models were obtained. The adequacy and significance of each model are summarized in Table S3 (Supplementary Materials). The summary of regression analysis results for measured responses for formula optimization is presented in Table S4 (Supplementary Materials). Moreover, the information derived from the models was expanded graphically by using isoresponsive curves. Figure 3 shows the response contour plots of the fitted model (3D plots). All plots were adjusted against X_1_ and X_3_ as they were defined as the most significant variables in this study, apart from gelation time.

Particularly, gelation time decreased with the increase of CaCl_2_·2H_2_O concentration and increased with increasing polymer concentration (either starch or alginate), as evidenced in Figure 3a,b. However, as represented in Table S4, none of the variables and interactions were considered significant in the tested concentration ranges (*p* > 0.05).

According to Kuo et al. [31], a higher concentration of polymer results in an increase in viscosity, leading to a slower rearrangement of the conformations of the polymer chain. This effect was observed, although not considered significant. In addition, previous studies have shown that a rapid gelation time leads to nonuniform crosslinking, which in turn can have a negative impact on the final properties of the product [19]. Hence, this type of parameter must be carefully evaluated, and for an adequate adjustment, it is necessary to tune the ratio CaCl_2_·2H_2_O–polymer(s) in function of the QTPP defined. Regarding construct integrity, the interaction X_1×3_ was disclosed as the most influencing term (*p* < 0.05) and revealed an antagonistic effect on the considered CQA (Figure 3c). In terms of spreading, none of the interactions under study had a distinct and significant impact on this parameter (Figure 3d). As observed in Figure 3e, the pore size increased with increasing starch percentage. The coefficient value revealed a synergetic impact on the X_3_ term. Furthermore, the overall results suggest that the addition of starch to an alginate-based 3D system can be advantageous for future drug release modulation strategies.

The formula critical parameters that were shown to affect gelation time, porosity, construct integrity, and spreadability were used to build the design space shown in Figure 3f. A broad and wider design space leads to a more robust and flexible formula. Every single point corresponds to a combination of starch content (X_3_) and percentage of CaCl_2_·2H_2_O (X_2_), with alginate (X_1_) defined as constant. This overlay plot provides a range within the values of a critical formula parameter that will not affect the final responses. From these results, it was possible to define the optimized formula, of which composition was the reflection of the target inputs. To guarantee a gelation time around 10–30 min, a porosity of 1–20 µm, and a structure with high precision, which involves the interaction between the construct integrity and the spreadability ratio, the Alg-St patch should be composed by 3% of alginate (factor contribution = 24.95), 2% of starch (factor contribution = 42.02), and 1.7% of CaCl_2_·2H_2_O as the crosslink solution (factor contribution = 33.03).

To statistically analyze the adequacy of the fitted models, an analysis of variance (ANOVA) was also performed. As shown in Table S3, the regression data shows that the fitted model for porosity responses is statistically significant (*p* < 0.05), highlighting the importance of the variables on the considered CQA. However, a significant model does not necessarily mean a correct explanation of the variation in results. The maximum square regression coefficient (R2) evaluates the model fit. Furthermore, the reproducibility is an additional parameter of analysis that infers over the variation of the replicates compared to overall variability, where the closer the value is to 1, the better is the fit. The reproducibility values obtained (>0.8) further support the sensitivity and the adequacy of the fitted models presented.

### 3.4. Effect of Starch on Physicochemical Properties of the 3D Alginate-Starch-Based Patches

Considering the outputs obtained from the response surface analysis and design space, the effects of starch on the physicochemical properties of the 3D Alg-St patches were analyzed in the optimized 3D Alg-St patch, using a 3D Alg-patch as the control.

**Figure 4:**
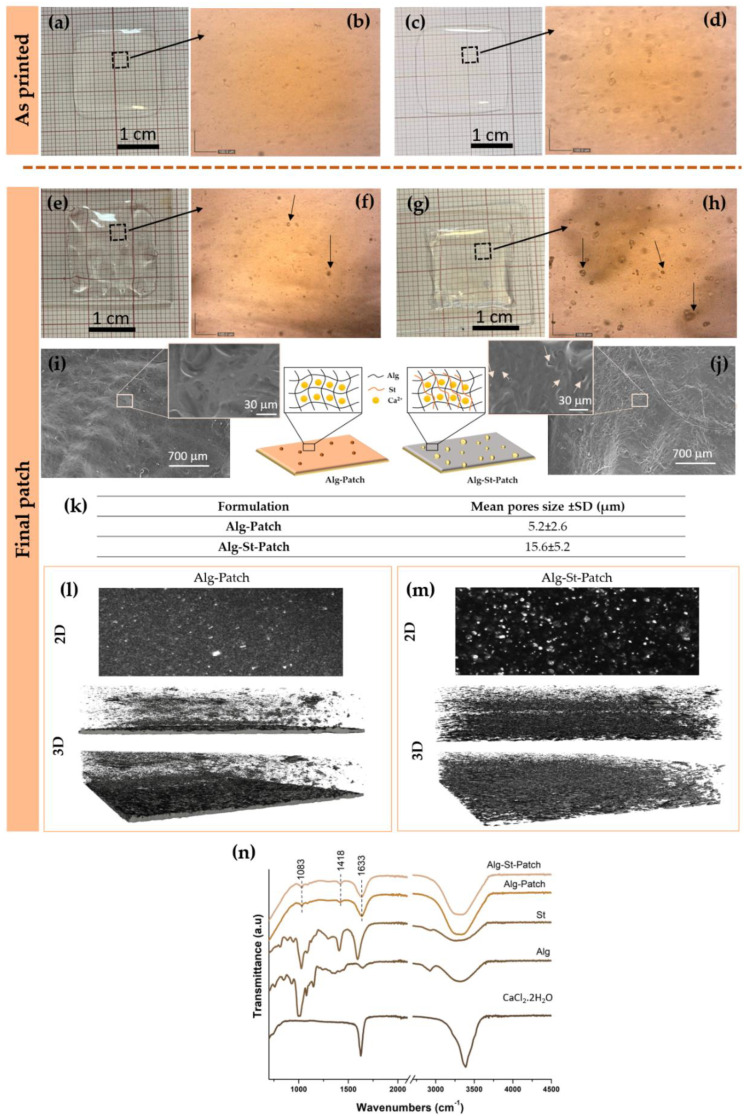
Effect of starch on the physicochemical properties of the 3D Alg and Alg-St patches before (as printed) and after crosslinking (final patch): Macroscopic and optical observations of Alg-Patch (**a**,**b**) and Alg-St-Patch (**c**,**d**) as printed; macroscopic and optical observations of Alg-Patch (**e**,**f**) and Alg-St-Patch (**g**,**h**) after crosslinking; scanning electron microscopy (SEM) images of Alg-Patch (**i**) and Alg-St-Patch (**j**) at 50× and 1000×; pore size distribution (**k**), Surface morphology, and topography of Alg-Patch (**l**) and Alg-St-Patch (**m**)—2D and 3D confocal data at 50×; and ATR-FTIR spectra of Alg and Alg-St patches, the crosslinker (CaCl_2_·2H_2_O), and alginate and starch powder (**n**).

The visual appearances of the 3D Alg-patch and 3D Alg-St-patch as printed (i.e., before adding CaCl_2_·2H_2_O crosslink solution) are shown in Figure 4. The 3D Alg-patch has a transparent slightly yellow color (Figure 4a), which changes to completely transparent after the addition of starch (Figure 4c). No significant modifications in patch geometry were observed in Alg-St-patch in comparison with Alg-patch (Figure 4a,c). However, to infer in detail the construct integrity and printability of the formulations, area values (mm^2^), printing accuracy (PA), and spreading ratios were determined and compared to the target. As printed, Alg-St-patch (419.9 ± 22.9; PA = 95.0%) presents an area closer to the target when compared to the Alg-patch (464.9 ± 16.1; PA = 83.8%) as well as a higher PA. The differences obtained can be attributed to the starch viscoelastic properties. Regarding the spreading ratio, similar values were obtained for Alg-St-patch (2.5 ± 0.6) and Alg-patch (3.0 ± 0.6). According to Freeman et al. [19], lower spreading ratios are desirable to allow the fabrication of hydrogel structures with high precision. Thus, the introduction of starch seems to only have a significant impact when increasing the size of the structure to be printed. The optical observation of both patches revealed that mixing starch with alginate increased the open porosity of the patch as shown in Figure 4b,d.

The visual appearances of the 3D Alg-patch and 3D Alg-St-patch after crosslinking (final patch) are shown in Figure 4. Macroscopic images of cross-linked Alg and Alg-St patches (Figure 4e,g, respectively), revealed a detachment of the lower part of the Alg-patch from the glass surface, while in Alg-St-patch, this effect was not visible. The observed detachment effect can be related to the gelation process. After cross-linking, Alg-patch showed better results in terms of area and PA (386.8 ± 6.2; PA = 96.7%) than the optimized Alg-St-patch (323.6 ± 7.5; PA = 80.9%). These results are opposite to the results obtained before gelation, which can be attributed to the fact that the Ca^2+^ ions have a greater affinity and higher ionic strength for link with alginate. Furthermore, during the ionic cross-linking gelation process, patches are subject to a contraction of the structure in volume, which is directly related to the crosslink concentration and the viscoelastic properties of the hydrogels. According to Kuo et al. [31], a high concentration of calcium ions results in shrinkage, while a low concentration results in swelling of the gel. However, the area dimensions obtained herein show that the addition of CaCl_2_·2H_2_O has an impact on the retraction of the structure regardless of its composition, since the percentage of retraction is proportional in both cases (i.e., when comparing the values for as printed and after crosslinking). In addition, the presence of free hydroxyl groups in the starch structure can improve water absorption capacity and can lead to an increase in the swelling degree of the hydrogel [8]. The results presented in Figure 4e,g reveal that the introduction of starch caused a significant retraction of the Alg-St-patch along the XY axis compared to the Alg-patch and an increase along the Z axis related to the swelling behavior. Regarding the spreading ratio, as already observed in the as printed patch, the Alg-St-patch (1.6 ± 0.5) showed similar values to the Alg-patch (2.4 ± 1.0).

To study in detail the influence of starch on the patch microstructure, optical and SEM imagens of the optimized 3D Alg-St-patch and the 3D Alg-patch were obtained, as shown in Figure 4. The images obtained by SEM were somewhat different from the optical observation. On the cross-linked patch surface of pure alginate, some filaments can be clearly observed, which interact with each other to form the network structure of the patch (Figure 4i). A similar network structure was observed on the Alg-St patch surface (Figure 4j). However, as already observed in the as printed patches (Figure 4b), the Alg-St-patch after gelation showed an evident increase in the size and quantity of open pores (Figure 4h,j,k). According to Ceylan Tuncaboylu et al. [33], when the matrix is composed solely of starch, decreasing its concentration does not change the size of the pores but decreases the pore wall thickness. This suggests that starch by itself does not increase the pore size of a matrix, but when encapsulated with other polymers, it may generate a structure reorganization which results in increased pore size. Additionally, according to the literature, calcium provides intermolecular interactions with more polar groups (–COO^−^), complicating the expansion of the polymeric chain which, consequently, loses flexibility, absorbing a low amount of water. On the contrary, the presence of starch leads to an increased swelling of the hydrogel due to the interaction of starch hydrophilic groups (–OH) with water molecules [8]. Thus, the differences observed in the porosity of the patches might be attributed to the interaction with water molecules during the gelation process. Keeping this in mind and considering that the gelation process can occur differently in Alg and Alg-St patches, 2D and 3D confocal microscope images were obtained after staining the hydrogel with silver nanoparticles (Figure 4l,m). The bright points on 2D confocal images were more intense and more frequent on Alg-St-patch (Figure 4m) than in Alg-patch (Figure 4l). Interestingly, this could be related to the number of silver nanoparticles retained on the open pores observed in optical and SEM images. Because the hydrogel networks are difficult to fully illustrate using 2D images, 3D volume images were also obtained. The 3D reconstruction revealed a less complex intricate network in the Alg-patch (Figure 4m) compared to the Alg-St-patch (Figure 4n). This suggests that the presence of starch can influence the internal network of the 3D patches.

**Figure 5:**
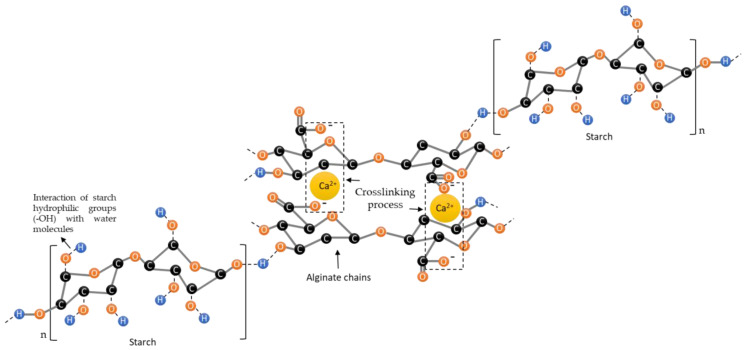
Representation of alginate and starch chains crosslinked with CaCl_2_·2H_2_O ions.

The functional groups of the Alg-patch and Alg-St-patch as well as the crosslinker (CaCl_2_·2H_2_O), alginate, and starch powder were investigated by ATR-FTIR spectroscopy, and the results are presented in Figure 4n. As observed in this figure, no visible differences were detected in the Alg-patch spectra when compared to Alg-St-patch. In both spectra, a small band at 1083 cm^−1^ representing the bending vibration of the C–O–C group was observed. Additionally, a small band at 1418 cm^−1^ and another more intense at 1620 cm^−1^ were identified and attributed to symmetric and asymmetric stretching of the carboxylate group −COO^−^, respectively [34,35]. The presence of these two bands suggests the involvement of COO^−^ groups in the Ca^2+^-mediated process of alginate reticulation [35]. Additionally, a broad strong band was noted in the region between 3200–3600 cm^−1^, which is related to the elongation of the −OH group of alginate [34]. Although the introduction of starch did not lead to significant differences at the structural level, additional data demonstrated that the variation in the percentage of CaCl_2_·2H_2_O caused changes in the detection of the bands (see the Supplementary Materials, Figure S3). Combining previously published data, with the data obtained herein, a schematic representation of alginate and starch chains crosslinked with CaCl_2_·2H_2_O ions is proposed (Figure 5) [8,36,37].

The results of these studies demonstrate that, by using the same alginate and crosslinker but by adding the starch to the hydrogel, it is possible to print constructs with different spatial microenvironments. Close control of the printed microenvironment can be extremely useful in the development of an efficient and controlled DDS.

#### Structural Stability of the 3D Alginate-Starch-Based Patches

**Figure 6:**
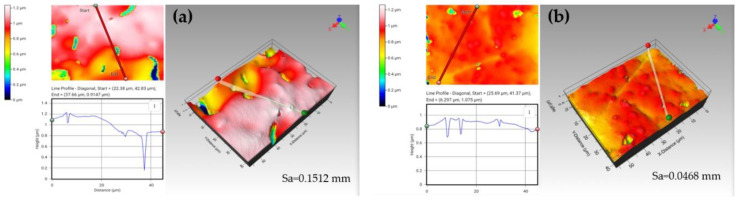
Optical profilometry images showing the bidimensional (2D) and tridimensional (3D) surface morphologies of (**a**) Alg-Patch and (**b**) Alg-St- Patch, the respective pore depth profiles, and roughness profile parameters.

Digitally processed bidimensional and tridimensional profilometric images, their correspondent pores depth profiles, and surface roughness (Sa) are shown in Figure 6. The results reveal clear morphological and topographic differences between the Alg-St-patch and Alg-patch. The Alg-patch presents deeper valleys and peaks (Figure 6a), while the Alg-St-Patch presents a more cohesive and homogeneous structure (Figure 6b), which is reflected in average surface roughness (Sa) results, with the Alg-Patch showing a higher Sa than Alg-St-Patch.

Macroscopic data also revealed that the Alg-patch underwent significant changes 7 days after printing, showing a decrease in height, suggesting a lower structural stability comparing to the Alg-St-patch. According to Córdoba et al. [16], the addition of starch to an alginate system can significantly decrease the alginate erosion features. Thus, the differences observed in the stability of the patches might be attributed to an erosion mechanism (i.e., mass loss from the matrix) during the 7 days of storage.

### 3.5. In Vitro Rhodamine B Release Studies from 3D Alginate-Starch-Based Patches

**Figure 7:**
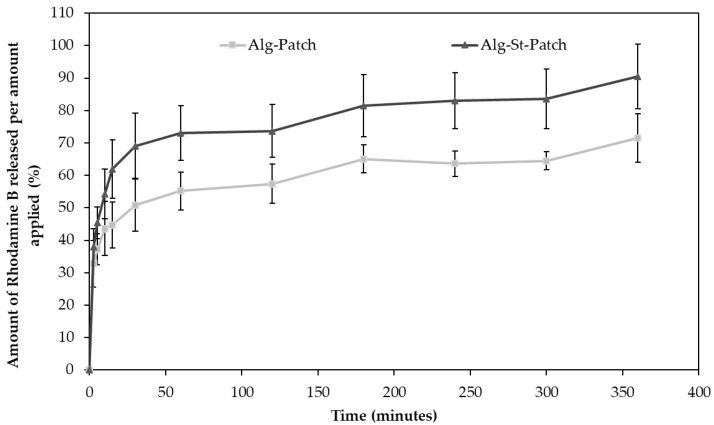
Release profiles of Rhodamine B from the Alg-patch and the Alg-St-patch (mean ± SD; *n* = 6).

The drug release rate from 3D patches is dependent on the drug solubility, influx of the medium into the structure, and polymer(s) swelling [38]. The release of the model drug Rhodamine B (RB) from the 3D patches matrices was faster in the first 30 min and slower afterwards, showing a burst effect possibly caused by rapid dissolution of freely soluble RB bound at the surface of the patches (Figure 7). The higher burst effect noticed on the Alg-St-patch can be attributed to the starch swelling properties. After 360 min, 71.51% of the Alg-patch drug content had been released whereas the optimized Alg-St-patch released 90.46% of the probe.

For the characterization of the RB release mechanism from the 3D patches, five different kinetic models were applied, i.e., zero-order, first-order, Higuchi, Korsmeyer–Peppas, and Weibull, and the fitting was evaluated by R^2^_adjusted_ and the AIC, which are the most widely used in the field of dissolution model.

For the control Alg-patch, the best fitting was obtained with the Korsmeyer–Peppas model (R^2^_adjusted_ = 0.962 ± 0.040), with slightly lower values for the Weibull model (R^2^_adjusted_ = 0.959 ± 0.047). Korsmeyer–Peppas is a useful mathematical model to study the drug release from hydrogel matrices when the release mechanism is not known or when more than one type of drug release phenomenon is involved. The Weibull model describes the phenomena and process associated to a finite time and is normally used to compare drug release profiles of matrix systems [39]. Comparing AIC values obtained for these two models, the Korsmeyer–Peppas showed a lower value, which corroborates the R^2^_adjusted_ results (see the Supplementary Materials, Table S5). Focusing on the Korsmeyer–Peppas model, the *n* value obtained was <0.5 (0.150), which suggests a Quasi–Fickian controlled release diffusion model. Regarding the optimized Alg-St-patch, the Weibull model exhibited a better fitting (R^2^_adjusted_ = 0.980 ± 0.006) than the Korsmeyer–Peppas model (R^2^_adjusted_ = 0.967 ± 0.008), and the obtained AIC values are close but distinct, with the Weibull model presenting a clear minimum. Focusing on this last model, the shape parameter (*β*) is 0.269, which characterizes the curve as parabolic (*β* < 1), with a higher initial slope and a subsequent exponential curve. Since both Korsmeyer–Peppas and Weibull models are good fits for both patches, their values can be easily compared. The *β* values obtained in the Weibull model for both patches were <1 (0.21 for Alg-patch and 0.269 for Alg-St-patch). According to Kosmidis et al. [40], *β* values below 0.35 are not found in simulation and experimental results. However, they may occur in highly disordered spaces much different than the percolation cluster [41]. In surface topography studies and SEM images, it was evident that the matrix had several irregularities and was not homogenous. They exhibit an irregular space, which can also be described as a fractal matrix. Bunde et al. [42] reported that “the nature of drug release drastically depends on the dimension of the matrix and is different depending on whether the matrix is a normal Euclidean space or a fractal material such as a polymer, corresponding to the fact that the basic laws of physics are quite different in a fractal environment”. On the other hand, the *n* values obtained with the Korsmeyer–Peppas model for both patches were <0.5 (0.150), which is beyond the limits of this model, suggesting a Quasi–Fickian controlled release diffusion model. According to the literature, this mechanism indicates that RB partially diffuses through a swollen matrix and water-filled pores in the 3D patches [43,44]. This information is in accordance with the combined release mechanism theory mentioned above (diffusion and swelling of the matrix). The obtained K_KP_, which is the release constant incorporating structural and geometric characteristics of the drug-dosage form, was 28.625 ± 4.744 for the Alg-patch, lower than the 37.154 ± 3.836 value obtained for the Alg-St-patch. The difference in the K_KP_ values of the formulations suggests a higher porosity in the Alg-St-patch, which may lead to higher permeability and, consequently, higher release rate. Moreover, pore size distribution and internal morphological analysis (Section 3.4) are in conformity with these observations.

## 4. Conclusions

Starch fulfils the general requirements for dermatological use. However, there is a significant lack of fundamental and basic research knowledge on starch-based inks for topical application. The overall results obtained herein showed that the addition of starch to an alginate-based 3D hydrogel patch led to a reorganization of the structure and to an increase of the size and quantity of open pores after crosslinking, showing higher peaks and valleys, which influence the drug diffusion release. Furthermore, the development of cost-effective DDS using 3D PAM printing technology might represent an opportunity to increase and personalize different therapeutic approaches. The versatility of the developed alginate-starch 3D patches makes them promising systems to be used for tailored-made drug delivery. Thus, the results obtained in this study provide an excellent baseline for future drug release modulation strategies.

## Figures and Tables

**Table 1:** Quality target product profile (QTPP) of the 3D hydrogel patch.

QTPP Element	Target
**Dosage form**	3D hydrogel patch
**Route of administration**	Topical
**Gelation time**	10–30 min [19]
**Porosity**	1–20 µm [20,21]
**Printing accuracy**	High [22]
**Elasticity**	Medium
**Adhesiveness**	High
**Biological effects**	Non-sensitizing and nonirritant [23]

**Table 2:** Risk Estimation Matrix (REM) presenting initial risk assessment levels of ionic cross-linked alginate-starch 3D patches for topical application, including formulation and process parameters. Low, low risk parameter; Medium, medium risk parameter; High, High risk parameter.

Failure Modes		3D Hydrogel Patch CQAs					
		Gelation Time	Porosity		Printing Accuracy	Elasticity	Adhesiveness
Raw Materials Attributes	Solvent (Water)	Amount	Medium	High	Medium	Medium	Low
	Gelation Agent (CaCl_2_·2H_2_O)	Amount	Medium	High	High	Medium	Low
	Polymer (Alginate/Starch)	Amount	Medium	High	High	Medium	Low
		Molecular weight	Medium	High	High	Medium	Low
		Viscosity	Medium	High	High	Medium	Low
	Drug	Solubility	Medium	Low	Low	Low	Low
		Amount	Medium	Low	Low	Low	Low
Process Parameters	Pre-manufacturing (Formulation)	Mixing time and rate	Medium	Medium	Medium	Low	Low
	Design and slicing	Printing setups	Medium	High	High	Low	Low
		Dimensions (x,y,z)	Medium	Medium	Medium	Low	Low
		Number of layers and contour strategy	Medium	Medium	Medium	Low	Low
		Feed rate	Low	Low	Low	Low	Low
	3D Printing	Needle material and diameter	Low	Low	Low	Low	Low
		Distance between the needle and the platform	Low	Low	Low	Low	Low
		Ink viscosity	Medium	High	Medium	High	High
		Surface adhesion materials	Low	Low	Medium	Low	Low
		Extrusion chamber filling	Low	Low	Low	Low	Low

## Supplementary Materials

The following are available online at https://www.mdpi.com/1999-4923/12/8/719/s1, Figure S1: Risk Estimation Matrix. Figure S2: 3D Alg-patch printed on a) agar-agar and b) glass support material. Figure S3: FTIR spectra of alginate and alginate-starch inks. P1 (Alginate 1.5% crosslinked with CaCl_2_ 0.7%); P2 (Alginate 4.5% crosslinked with CaCl_2_ 0.7%); P3 (Alginate 1.5% crosslinked with CaCl_2_ 3%); P4 (Alginate 4.5% crosslinked with CaCl_2_ 3%); P5 (Alginate(1.5%)-Starch(4%) crosslinked with CaCl_2_ 0.7%); Alg (Alginic acid sodium salt powder); CaCl_2_ (Calcium chloride powder); St (INSTANT PURE-COTE® B793 modified starch powder). Table S1: Design of experiments (Alg-St 3D hydrogel patch optimization). Table S2: REM presenting initial risk assessment levels of ionic cross-linked alginate-starch 3D patches for topical application, including formulation and process parameters. S, severity; O, Occurrence; D, Detectability; RPN, Risk Priority Number. Table S3: ANOVA parameter summary of fitted model’s characterization. Table S4: Summary of regression analysis results for measured responses (Full Factorial Design composed by 3 levels), for formula optimization. Table S5: Parameters obtained by fitting 5 different kinetic models to the release data from the 3D patches (mean ± SD; *n* = 5).

## References

[1] Ventola C.L. (2014). Medical Applications for 3D printing: Current and projected uses. P T.

[2] Konta A.A., García-Piña M., Serrano D.R. (2017). Personalised 3D printed medicines: Which techniques and polymers are more successful?. Bioengineering.

[3] Economidou S.N., Lamprou D.A., Douroumis D. (2018). 3D printing applications for transdermal drug delivery. Int. J. Pharm..

[4] Goyanes A., Amornrat D.U., Wang J., Basit A.W., Gaisford S. (2016). 3D scanning and 3D printing as innovative technologies for fabricating personalized topical drug delivery systems. J. Control. Release.

[5] Azad M.A., Olawuni D., Kimbell G., Badruddoza A.Z.M., Hossain M.S., Sultana T. (2020). Polymers for extrusion-based 3D printing of pharmaceuticals: A holistic materials–process perspective. Pharmaceutics.

[6] Liu Z., Jiang Q., Zhang Y., Li T., Zhang H.C. Sustainability of 3D printing: A critical review and recommendations. Proceedings of the ASME 2016 11th International Manufacturing Science and Engineering Conference.

[7] Demirtaş T.T., Irmak G., Gümüşderelioǧlu M. (2017). A bioprintable form of chitosan hydrogel for bone tissue engineering. Biofabrication.

[8] Fernandes R.d.S., Tanaka F.N., Moura M.d.R., Aouada F.A. (2019). Development of alginate/starch-based hydrogels crosslinked with different ions: Hydrophilic, kinetic and spectroscopic properties. Mater. Today Commun..

[9] Azeredo H.M.C., Rosa M.F., De Sá M., Souza Filho M., Waldron K., Waldron K.W. (2014). The use of biomass for packaging films and coatings. Advances in Biorefineries: Biomass and Waste Supply Chain Exploitation.

[10] Koski C., Onuike B., Bandyopadhyay A., Bose S. (2018). Starch-hydroxyapatite composite bone scaffold fabrication utilizing a slurry extrusion-based solid freeform fabricator. Addit. Manuf..

[11] Chen H., Xie F., Chen L., Zheng B. (2019). Effect of rheological properties of potato, rice and corn starches on their hot-extrusion 3D printing behaviors. J. Food Eng..

[12] Maniglia B.C., Lima D.C., Matta Junior M.D., Le-Bail P., Le-Bail A., Augusto P.E.D. (2019). Hydrogels based on ozonated cassava starch: Effect of ozone processing and gelatinization conditions on enhancing 3D-printing applications. Int. J. Biol. Macromol..

[13] Yang F., Zhang M., Bhandari B., Liu Y. (2018). Investigation on lemon juice gel as food material for 3D printing and optimization of printing parameters. LWT Food Sci. Technol..

[14] Zheng L., Yu Y., Tong Z., Zou Q., Han S., Jiang H. (2019). The characteristics of starch gels molded by 3D printing. J. Food Process. Preserv..

[15] Singh B., Sharma D.K., Kumar R., Gupta A. (2009). Controlled release of the fungicide thiram from starch-alginate-clay based formulation. Appl. Clay Sci..

[16] Córdoba A.L., Deladino L., Martino M. (2013). Effect of starch filler on calcium-alginate hydrogels loaded with yerba mate antioxidants. Carbohydr. Polym..

[17] Hosseini S.M., Hosseini H., Mohammadifar M.A., German J.B., Mortazavian A.M., Mohammadi A., Darani K.K., Aliabadi S.S., Khaksar R. (2014). Preparation and characterization of alginate and alginate-resistant starch microparticles containing nisin. Carbohydr. Polym..

[18] Vazquez G.L., Calleros C.L., Buendia H.E., Chavez G., Ramirez J.A., Carter E.J.V. (2015). Effect of the weight ratio of alginate-modified tapioca starch on the physicochemical properties and release kinetics of chlorogenic acid containing beads. Food Hydrocoll..

[19] Freeman F.E., Kelly D.J. (2017). Tuning alginate bioink stiffness and composition for controlled growth factor delivery and to spatially direct MSC Fate within bioprinted tissues. Sci. Rep..

[20] Sujka M., Jamroz J. (2010). Characteristics of pores in native and hydrolyzed starch granules. Starch Staerke.

[21] Simpliciano C., Clark L., Asi B., Chu N., Mercado M., Diaz S., Goedert M., Mobed-Miremadi M. (2013). Cross-linked alginate film pore size determination using atomic force microscopy and validation using diffusivity determinations. J. Surf. Eng. Mater. Adv. Technol..

[22] He Y., Yang F., Zhao H., Gao Q., Xia B., Fu J. (2016). Research on the printability of hydrogels in 3D bioprinting. Sci. Rep..

[23] Marto J., Pinto P., Fitas M., Gonçalves L.M., Almeida A.J., Ribeiro H.M. (2018). Safety assessment of starch-based personal care products: Nanocapsules and pickering emulsions. Toxicol. Appl. Pharm..

[24] Zhang Y., Huo M., Zhou J., Zou A., Li W., Yao C., Xie S. (2010). DDSolver: An add-in program for modeling and comparison of drug dissolution profiles. AAPS J..

[25] Marto J., Gouveia L.F., Gonçalves L.M., Gaspar D.P., Pinto P., Carvalho F.A., Oliveira E., Ribeiro H.M., Almeida A.J. (2016). A quality by design (QbD) approach on starch-based nanocapsules: A promising platform for topical drug delivery. Colloids Surf. B Biointerfaces.

[26] Cohen D.L., Tsavaris A.M., Lo W.M., Bonassar L.J., Lipson H. (2008). Improved quality of 3D-printed tissue constructs through enhanced mixing of alginate hydrogels. Res. Gate.

[27] Piskounova S., Rojas R., Bergman K., Hilborn J. (2011). The effect of mixing on the mechanical properties of hyaluronan-based injectable hydrogels. Macromol. Mater. Eng..

[28] Guo T.R., Holzber T.G., Lim C., Gao F., Gargava A.E., Trachtenberg J.G., Mikos A.P., Fisher J. (2017). 3D printing PLGA: A quantitative examination of the effects of polymer composition and printing parameters on print resolution. Biofabrication.

[29] Farzadi A., Waran V., Solati-Hashjin M., Rahman Z.A.A., Asadi M., Osman N.A.A. (2015). Effect of layer printing delay on mechanical properties and dimensional accuracy of 3D printed porous prototypes in bone tissue engineering. Ceram. Int..

[30] Farrés I.F., Norton I.T. (2014). Formation kinetics and rheology of alginate fluid gels produced by in-situ calcium release. Food Hydrocoll..

[31] Kuo C.K., Ma P.X. (2001). Ionically crosslinked alginate hydrogels as scaffolds for tissue engineering: Part 1. Structure, gelation rate and mechanical properties. Biomaterials.

[32] Viyoch J., Sudedmark T., Srema W., Suwongkrua W. (2005). Development of hydrogel patch for controlled release of alpha-hydroxy acid contained in tamarind fruit pulp extract. Int. J. Cosmet. Sci..

[33] Ceylan Tuncaboylu D., Abdurrahmanoglu S., Gazioglu I. (2019). Rheological characterization of starch gels: A biomass based sorbent for removal of polycyclic aromatic hydrocarbons (PAHs). J. Hazard. Mater..

[34] Zhu B., Ma D., Wang J., Zhang S. (2015). Structure and properties of semi-interpenetrating network hydrogel based on starch. Carbohydr. Polym..

[35] Voo W.P., Lee B.B., Idris A., Islam A., Tey B.T., Chan E.S. (2015). Production of ultra-high concentration calcium alginate beads with prolonged dissolution profile. RSC Adv..

[36] Costa M.J., Marques A.M., Pastrana L.M., Teixeira J.A., Sillankorva S.M., Cerqueira M.A. (2018). Physicochemical properties of alginate-based films: Effect of ionic crosslinking and mannuronic and guluronic acid ratio. Food Hydrocoll..

[37] Onyido I., Sha’Ato R., Nnamonu L.A. (2012). Environmentally friendly formulations of trifluralin based on alginate modified starch. J. Environ. Prot..

[38] Szekalska M., Sosnowska K., Kósnik A.C., Winnicka K. (2018). Calcium chloride modified alginate microparticles formulated by the spray drying process: A strategy to prolong the release of freely soluble drugs. Materials.

[39] Bruschi M.L., Bruschi M.L. (2015). Mathematical models of drug release. Strategies to Modify Drug Release from Pharmaceutical Systems.

[40] Kosmidis K., Argyrakis P., Macheras P. (2003). Fractal kinetics in drug release from finite fractal matrices. J. Chem. Phys..

[41] Papadopoulou V., Kosmidis K., Vlachou M., Macheras P. (2006). On the use of the Weibull function for the discernment of drug release mechanisms. Int. J. Pharm..

[42] Bunde A., Havlin S., Nossal R., Stanley H.E., Weiss G.H. (1985). On controlled diffusion-limited drug release from a leaky matrix. J. Chem. Phys..

[43] Cojocaru V., Ranetti A.E., Hinescu L.G., Ionescu M., Cosmescu C., Poștoarcă A.G., Cinteză L.O. (2015). Formulation and evaluation of in vitro release kinetics of na3cadtpa decorporation agent embedded in microemulsion-based gel formulation for topical delivery. Farmacia.

[44] Gupta B., Chaurasia U., Chakraborty P. (2014). Design and development of oral transmucosal film for delivery of salbutamol sulphate. J. Pharm. Chem. Biol. Sci..

