# Supplementary material for: Effects of Starch Incorporation on the Physicochemical Properties and Release Kinetics of Alginate-Based 3D Hydrogel Patches for Topical Delivery
Source: Pharmaceutics. 2020 Jul 31;12(8):719. doi: 10.3390/pharmaceutics12080719 (PMC7466037; doi:10.3390/pharmaceutics12080719)
Supplement: Supplementary file 1 [file pharmaceutics-12-00719-s001.pdf]

pharmaceutics

Supplementary Materials: Effects of Starch Incorporation on the Physicochemical Properties and Release Kinetics of Alginate-Based 3D Hydrogel Patches for Topical Delivery

Sara Bom, Catarina Santos, Rita Barros, Ana M. Martins, Patrizia Paradiso, Ricardo Cláudio, Pedro Contreiras Pinto, Helena M. Ribeiro and Joana Marto *

Methods

Risk Estimation Matrix (REM)

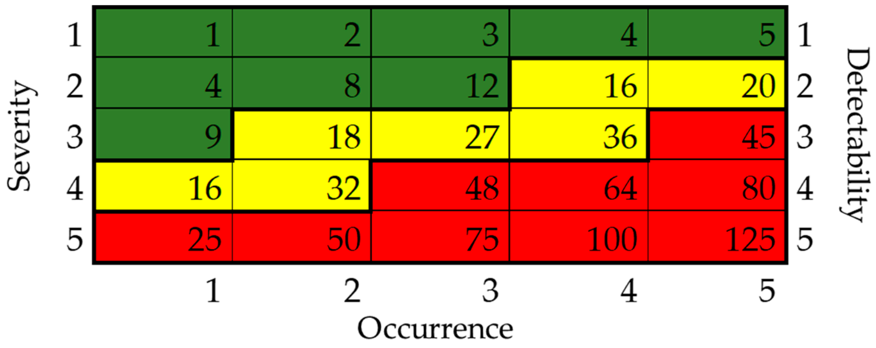

Figure S1. Risk Estimation Matrix.

Formula optimization and establishment of Design Space

pharmaceutics MDPI

Table S1. Design of Experiments (Alg-St 3D Hydrogel Patch Optimization).

Exp Name	Run Order	Design Matrix			Experimental Matrix for Formula Optimization (%)			
		Alginate	Starch	$CaCl_2.2H_2O$	Alginate	Starch	Solvent (H_2O)	$CaCl_2.2H_2O$
**N1**	5	-1	-1	-1	1.5	0	98.5	0.7
**N2**	6	1	-1	-1	4.5	0	95.5	0.7
**N3**	2	-1	-1	1	1.5	0	98.5	3
**N4**	9	1	-1	1	4.5	0	95.5	3
**N5**	1	-1	1	-1	1.5	4	95.5	0.7
**N6**	3	1	1	-1	4.5	4	91.5	0.7
**N7**	11	-1	1	1	1.5	4	95.5	3
**N8**	7	1	1	1	4.5	4	91.5	3
**N9**	4	0	0	0	3	2	95.0	1.85
**N10**	10	0	0	0	3	2	95.0	1.85
**N11**	8	0	0	0	3	2	95.0	1.85

Results

Adjustment of CPPs

Among the various support materials studied to improve adhesion during patch printing, glass resulted in more accurate printing (Figure S1).

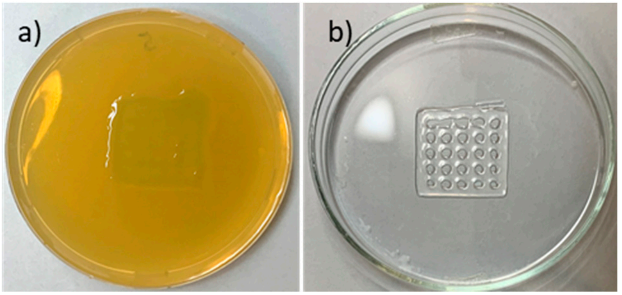

Figure S2. 3D Alg-patch printed on **a**) agar-agar and **b**) glass support material.

pharmaceutics

MDPI

Formula Optimization and Establishment of Design Space

Table S2. REM presenting initial risk assessment levels of ionic cross-linked alginate-starch 3D patches for topical application, including formulation and process parameters. S, severity; O, Occurrence; D, Detectability; RPN, Risk Priority Number.

Failure Modes			Gelation Time				Porosity				Printing Accuracy				Elasticity				Adhesiveness			
			**S**	**O**	**D**	**RPN**	**S**	**O**	**D**	**RPN**	**S**	**O**	**D**	**RPN**	**S**	**O**	**D**	**RPN**	**S**	**O**	**D**	**RPN**
**CMAs**	Solvent (Water)	Amount	4	3	2	24	4	3	4	48	4	3	2	24	4	2	3	24	2	1	3	6
	Gelation Agent ($CaCl_2.2H_2O$)	Amount	5	3	3	45	4	3	4	48	5	4	3	60	4	3	3	36	2	2	4	16
	Polymer (Alginate/Starch)	Amount	5	3	3	45	4	3	4	48	5	4	3	60	4	3	3	36	2	2	4	16
		Molecular weight	5	3	3	45	4	3	4	48	5	4	3	60	4	3	3	36	2	2	4	16
		Viscosity	5	3	3	45	4	3	4	48	5	4	3	60	4	3	3	36	2	2	4	16
	Drug	Solubility	4	3	2	24	2	1	3	6	3	2	3	18	1	2	3	6	1	2	3	6
		Amount	4	3	2	24	2	1	3	6	3	2	3	18	1	2	3	6	1	2	3	6
**CPPs**	Pre-manufacturing (Formulation)	Mixing time and rate	4	2	3	24	3	2	4	24	3	2	4	24	2	2	2	8	2	2	2	8
	Design and slicing	Printing setups	4	2	3	24	4	4	4	64	4	4	4	64	2	2	3	12	2	2	3	12
		Dimensions (x,y,z)	5	3	2	30	3	3	3	27	5	3	2	30	2	2	2	8	2	2	2	8
		Number of layers and contour strategy	5	3	2	30	3	3	3	27	5	3	2	30	3	2	2	12	3	2	2	12
		Feed rate	2	2	2	8	4	3	4	48	5	3	3	45	2	1	3	6	2	1	3	6
	3D Printing	Needle material and diameter	2	2	2	8	4	2	3	24	5	3	2	30	2	1	3	6	2	1	3	6
		Distance between the needle and the platform	2	1	1	2	2	1	1	2	5	2	2	20	2	1	3	6	2	1	3	6
		Ink viscosity	5	3	2	30	5	3	4	60	5	3	2	30	4	3	4	48	4	3	4	48
		Surface adhesion materials	1	1	1	1	2	2	5	20	5	3	2	30	2	1	3	6	2	1	3	6
		Extrusion chamber filling	2	2	2	8	2	2	4	16	4	2	2	16	2	1	3	6	2	1	3	6

Table S3. ANOVA parameter summary of fitted model's characterization.

CQAs	Regression				Lack of Fit	
	F	*p*	R^2	Reproducibility	F	*p*
Gelation Time	1.683	0.320	0.716	0.992	178.944	0.006
Construct Integrity	2.377	0.211	0.781	1.000	2842.070	0.000
Spreadability	1.194	0.452	0.642	0.800	7.116	0.123
Porosity	8.839	0.027	0.930	0.976	13.879	0.067

Table S4. Summary of regression analysis results for measured responses (Full Factorial Design composed by 3 levels), for formula optimization.

CQAs	Regression	β_0	β_1	β_2	β_3	β_{12}	β_{13}	β_{23}
Gelation Time	Coeff	16.045	1.181	-2.632	2.086	0.388	-0.436	-0.654
	± SE	1.108	1.162	1.162	1.162	1.039	1.039	1.039
	p	0.0001	0.3671	0.0862	0.1471	0.7279	0.6964	0.5633
Construct Integrity	Coeff	314.142	16.252	-17.681	0.141	17.128	-31.624	-12.142
	± SE	12.310	12.911	12.911	12.911	11.547	11.547	11.547
	p	<0.0001	0.2766	0.2427	0.9918	0.2122	0.0419	0.3524
Spreadability	Coeff	2.881	-0.218	-0.070	-0.247	-0.145	0.009	-0.215
	± SE	0.158	0.166	0.166	0.166	0.148	0.148	0.148
	p	<0.0001	0.2597	0.6934	0.2112	0.3845	0.9546	0.2215
Porosity	Coeff	9.873	-0.568	0.353	3.515	0.002	-0.514	-0.066
	± SE	0.475	0.497	0.497	0.497	0.445	-0.445	0.445
	p	<0.0001	0.3175	0.5170	0.0021	0.9966	0.3125	0.8893

Effect of starch and crosslink solution on the 3D alginate-starch-based patches (ATR-FTIR)

Attenuated total reflection Fourier-transform IR spectroscopy (ATR-FTIR) was used to examine which chemical bonds were present in the two polymers system. The ATR-FTIR spectra of 5 patches, and the correspondent raw materials powders, are shown in Figure S2. The results obtained from patches P1 to P4 show that an increase in the alginate percentage from 1.5% to 4.5% did not cause significant changes in the vibrations of the bands. However, an increase in the $CaCl_2$ percentage from 0.7% to 3%, significantly altered the ATR-FTIR spectra, namely with the intensification of the bands at 1031, 1086, 1125 and 1418 cm^{-1}, and the emergence of four new bands at 940, 994, 1300 and 1598 cm^{-1}. The results clearly evidence that an increase of $CaCl_2$ concentration strengthens the asymmetric elongation of carbonate groups, as well as the C–O–C and C-O-H stretching vibrations [1, 2]. The comparison of spectra for the P3 and P5 patches, suggests that the presence of starch in the structural composition caused the disappearance of some vibrations, namely the bands at 935, 947 and at 1598 cm^{-1} that were visible in patch P3, without starch. In addition, there was a slight deviation in the wave number of some of the peaks. Together, these results suggest that the addition of starch weakened the intermolecular bond between chains, which can have an impact on the molecular structure of the printed patches.

pharmaceutics

MDPI

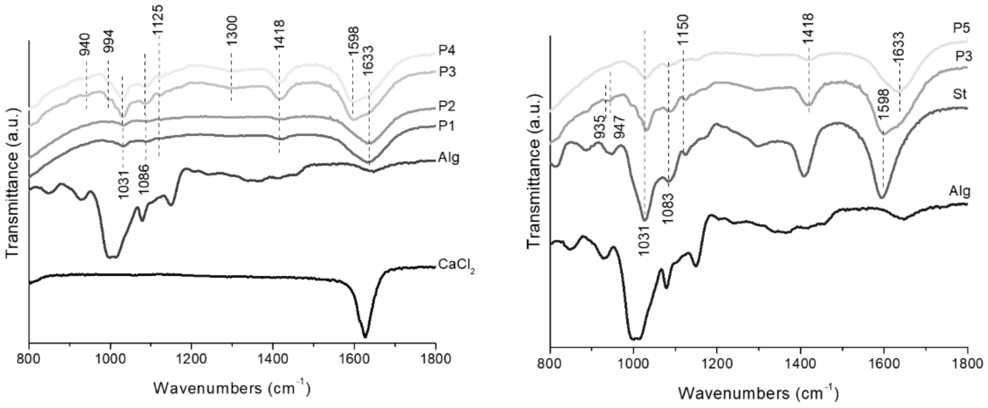

Figure S3. FTIR spectra of alginate and alginate-starch inks. P1 (Alginate 1.5% crosslinked with $CaCl_2$ 0.7%); P2 (Alginate 4.5% crosslinked with $CaCl_2$ 0.7%); P3 (Alginate 1.5% crosslinked with $CaCl_2$ 3%); P4 (Alginate 4.5% crosslinked with $CaCl_2$ 3%); P5 (Alginate(1.5%)-Starch(4%) crosslinked with $CaCl_2$ 0.7%); Alg (Alginic acid sodium salt powder); $CaCl_2$ (Calcium chloride powder); St (INSTANT PURE-COTE® B793 modified starch powder).

In Vitro Rhodamine B Release Studies from 3D Alginate-Starch-Based Patches

Table S5. Parameters obtained by fitting 5 different kinetic models to the release data from the 3D patches (mean ± SD; *n* = 5).

Formulation	Model	K	$R^2_{adjusted}$	AIC
Alg-Patch	Zero order	0.257 ± 0.016	-1.328 ± 0.534	112,377 ± 1.747
	First order	0.007 ± 0.002	-0.632 ± 0.520	107.895 ± 2.336
	Higuchi	4.556 ± 0.259	0.013 ± 0.361	101.750 ± 2.958
	Korsmeyer-Peppas	28.625 ± 4.744<br>n-0.150 ± 0.032	0.962 ± 0.040	59.960 ± 10.167
	Weibull	α-3.124 ± 0.679<br>β-0.21 ± 0.052<br>Ti-0.849 ± 0.840	0.959 ± 0.047	61.201 ± 10.588
Alg-St-Patch	Zero order	0.332 ± 0.035	-1.373 ± 0.163	119.140 ± 2.744
	First order	0.066 ± 0.024	0.432 ± 0.475	98.735 ± 7.090
	Higuchi	5.933 ± 0.636	0.013 ± 0.088	108.597 ± 2.724
	Korsmeyer-Peppas	37.154 ± 3.836<br>n-0.150 ± 0.008	0.9665 ± 0.008	68.5605 ± 5.9503
	Weibull	α-2.327 ± 0.219<br>β-0.269 ± 0.053<br>Ti-1.371 ± 0.489	0.9798 ± 0.006	63.120 ± 5.210

References:

1. Zhu, B.; Ma, D.; Wang, J.; Zhang, S. Structure and properties of semi-interpenetrating network hydrogel based on starch. *Carbohydr. Polym.* **2015**, *133*, 448–455, doi:10.1016/j.carbpol.2015.07.037.

2. Voo, W.P.; Lee, B.B.; Idris, A.; Islam, A.; Tey, B.T.; Chan, E.S. Production of ultra-high concentration calcium alginate beads with prolonged dissolution profile. *RSC Adv.* **2015**, *5*, 36687–36695, doi:10.1039/c5ra03862f.
